# Assessment of microbial quality in poultry drinking water on farms in Austria

**DOI:** 10.3389/fvets.2023.1254442

**Published:** 2023-11-22

**Authors:** Azra Mustedanagic, Monika Matt, Karin Weyermair, Anna Schrattenecker, Isabella Kubitza, Clair L. Firth, Igor Loncaric, Martin Wagner, Beatrix Stessl

**Affiliations:** ^1^FFoQSI GmbH-Austrian Competence Centre for Feed and Food Quality, Safety and Innovation, Tulln, Austria; ^2^Unit of Food Microbiology, Institute of Food Safety, Food Technology and Veterinary Public Health, University of Veterinary Medicine Vienna, Vienna, Austria; ^3^Department of Statistics and Analytical Epidemiology, Austrian Agency for Health and Food Safety (AGES), Innsbruck, Austria; ^4^Department of Statistics and Analytical Epidemiology, Austrian Agency for Health and Food Safety (AGES), Graz, Austria; ^5^Unit of Veterinary Public Health and Epidemiology, Institute of Food Safety, Food Technology and Veterinary Public Health, University of Veterinary Medicine Vienna, Vienna, Austria; ^6^Institute of Microbiology, Department of Pathobiology, University of Veterinary Medicine Vienna, Vienna, Austria

**Keywords:** water line treatment, opportunistic pathogens, poultry health, *Pseudomonas*, antimicrobial susceptibility

## Abstract

The quality of poultry drinking water has a significant effect on broiler health and performance. This study conducted an analysis of aerobic mesophilic counts (AMC), *Enterobacteriaceae* (EB), *Pseudomonadaceae* (PS), and screened for the presence of *Campylobacter* spp. in water samples collected from a total of 14 farms in Austria, with either a public or private water source. The efficacy of two water line treatment methods was evaluated: a chemical treatment of the water lines with 4.0 ppm ClO_2_ (T1) and a combined chemical (4.0 ppm active ClO_2_ and 3.0% peracetic acid) and mechanical treatment (purging of the water lines with a high-pressure air pump; T2). However, both the T1 and T2 treatments failed to reduce the AMC counts below the maximum acceptable microbial limit of 4.0 log_10_ CFU/ml in water samples. In addition, no significant reduction in EB and PS counts was observed in water samples after either T1 or T2 water line treatment. The water samples showed a high level of microbial diversity with 18 to 26 different genera. The genus *Pseudomonas* was most frequently isolated across all poultry farms, while *Campylobacter jejuni* was identified in a single sample collected before water line treatment. Isolate analysis revealed the presence of opportunistic pathogens in water samples both before (T1 43.1%, T2 30.9%) and after (T1 36.3%, T2 33.3%) water line treatment. Opportunistic pathogens belonging to genera including *Pseudomonas* spp., *Stenotrophomonas* spp., and *Ochrobactrum* spp., were most frequently isolated from poultry drinking water. These isolates exhibited multidrug resistance and resistance phenotypes to antimicrobials commonly used in Austrian poultry farms. The findings of this study emphasize the potential risk of exposure to opportunistic pathogens for poultry and personnel, underscoring the importance of efficient water line management.

## 1 Introduction

Poultry is one of the main sources of meat production worldwide (1). In 2020, more than 97 million chickens were processed in Austria, representing 124.000 tons of processed poultry meat (2). Drinking water is a vital nutrient for commercial poultry and has a significant impact on poultry health, liveweight, feed conversion ratios, and overall performance (3, 4). The water consumption of poultry is approximately twice the amount of feed intake (5). Poultry health and water intake are directly influenced by microbial water quality (4, 6, 7).

In Europe, the water quality standards for poultry drinking water have been adapted from water quality regulations intended for human drinking water consumption (8), EC Directive 98/83/EC (Drinking Water Directive [DWD] 9). According to the Austrian Poultry Hygiene Regulation (10) drinking water used for poultry production must not exceed a total aerobic mesophilic count (AMC) of 2.0 log_10_ and 1.3 log_10_ colony forming units (CFU/ml) at 22° and 37°C, respectively. Currently, there is no legal requirement to examine microbial contamination inside the drinking water lines (11). Hence, maintenance of water line hygiene is primarily the responsibility of the poultry producer, and it is typically conducted between the production cycles (12). The standard water line practices involve mechanical cleaning by flushing the water lines, followed by oxidative disinfection, primarily using chlorination or acidifiers (7, 12–14).

While water line treatment is a crucial component of an effective biosecurity program, its effectiveness does not ensure the complete elimination of the microorganisms within the water lines (15–17). *Escherichia coli*, *Salmonella* spp., and *Campylobacter* spp. have been detected in poultry drinking water (7, 18). Elevated temperatures and low water flow rates in enclosed water line systems have been found to adversely affect water quality, as indicated by previous studies (4, 12). These conditions are favorable for the accumulation of dissolved organic substances, minerals, and solid particles, which facilitate growth and promote the formation of biofilms. Among biofilm-forming bacteria, primarily *Pseudomonas* and *Stenotrophomonas* are responsible for biofilm formation on surfaces of poultry drinking lines (12). Biofilms may provide a favorable surface for attachment of opportunistic pathogens (OP), such as such as *Acinetobacter*, *Aeromonas, Citrobacter, Enterobacter,* and *Klebsiella* whose members are natural inhabitants of plumbing systems and adapted to survival in drinking water (19). Although these bacteria are generally not pathogenic, some have the potential to cause infections in susceptible poultry and farm workers (20). Hence, the detachment of pathogen and OP rich biofilms and their contamination of the water system present a significant risk for waterborne transmission of these bacteria, posing a potential threat to both poultry and human health. Moreover, the administration of medication to poultry through drinking water, which is a preferred route, has been linked to presence of multidrugresistant (MDR) bacteria (21, 22).

Microbial water quality is frequently evaluated at its source, but assessments at the end of the drinking lines are infrequent, despite the potential for substantial variations in microbial quality between the source and endpoint (12). Thus, the objective of this study was to evaluate the microbial quality of water samples collected at the end of a production cycle of five to six weeks and shortly before restocking for the subsequent production cycle, following the water line treatment. Previous studies have demonstrated the presence of pathogens such as *Campylobacter* spp. in poultry water on farms with private water supplies compared to those with a public supply (23, 24). This highlights the critical role of poultry drinking water as a potential source of *Campylobacter* spp. infection on the farm (25, 26). The presence of *Campylobacter* spp. in drinking water on poultry farms may indicate lapses in biosecurity, contaminated water source, ineffective and/or incorrectly applied water line cleaning procedures (11, 18). Therefore, one of our objectives was to assess the microbial quality of poultry drinking water in farms with either public or private water supply. We applied ISObased reference methods to assess bacterial load and presence of *Campylobacter* spp. in poultry drinking water, followed by partial 16S rRNA sequencing of bacterial isolates. Antibiotic susceptibility patterns of commonly isolated OP were then determined.

## 2 Materials and methods

### 2.1 Water line treatment and sample collection

**Figure 1:**
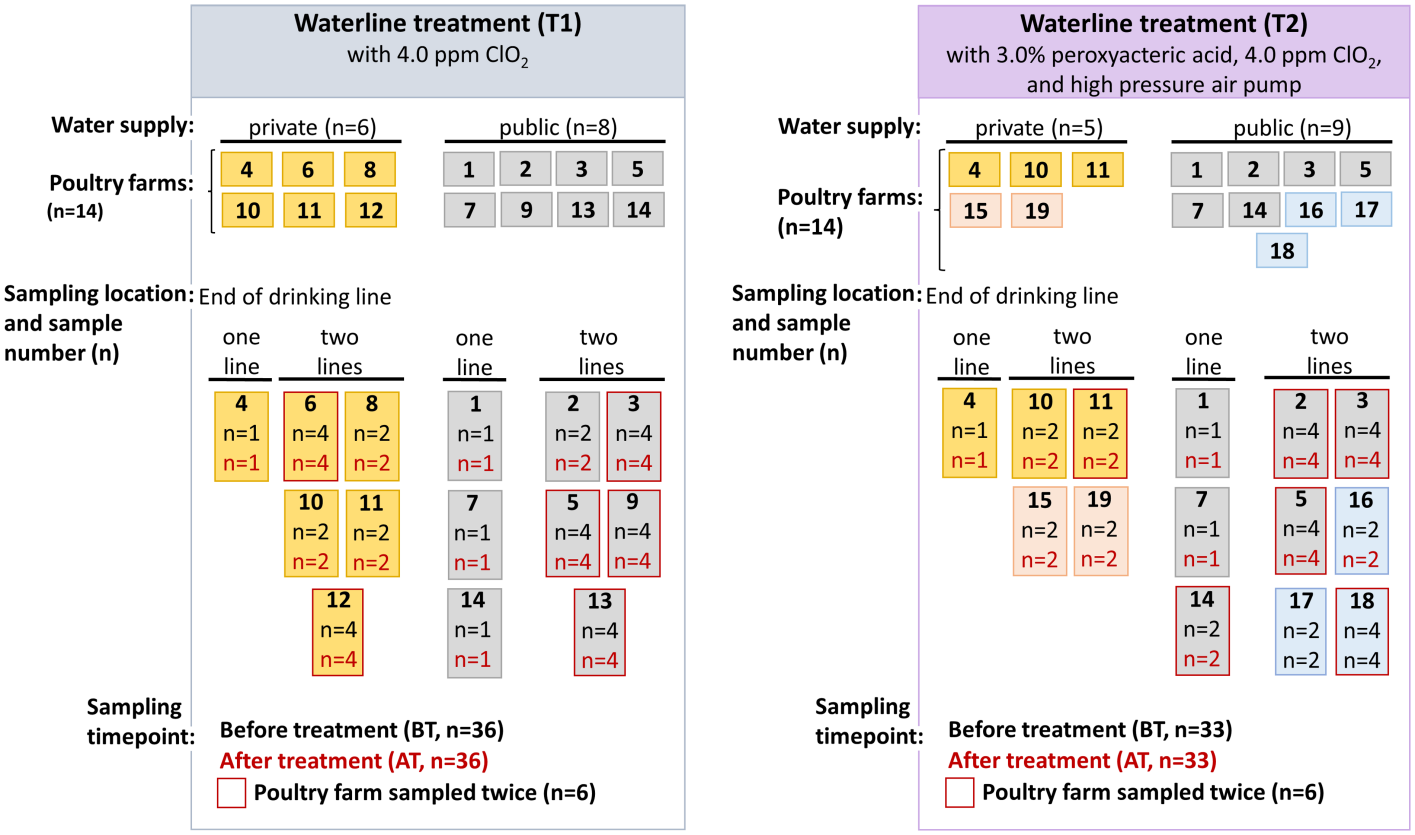
An overview of the sampling conducted in fourteen poultry farms during chemical (T1) and combined chemical with mechanical (T2) water line treatment. Poultry farms 6, 8, 9, 12, and 13 withdrew their participation after T1 and were substituted by the poultry farms 15 and 19 (indicated by pink color) with private water supply, and 16–18 (indicated blue color) with public water supply during T2 water line sampling.

Twenty-eight poultry farms producing broilers for local slaughterhouses in Austria voluntarily participated in the study between May 2019 and August 2020, some of which had private (*n* = 11) and others public (*n* = 17) water supplies. The fattening period at the participating poultry farms in Austria was five to six weeks. The poultry farms were divided into two distinct groups based on whether the farms employed solely chemical (T1) or a combination of chemical and mechanical (T2) water line treatment methods. An overview of the poultry farms included in the study is presented in Figure 1. Cleaning and water line treatment at the poultry farms was performed by the farmer. Since the participation of poultry farms in the study was voluntary, poultry farms 6, 8, 9, 12, and 13 withdrew their participation after T1 and were substituted by the poultry farms 15–19 during T2. The study was conducted in collaboration with a private laboratory (HYGIENICUM GmbH, Graz, Austria), which provided training on the water line cleaning procedures to be implemented at the poultry farms to the participating farmers.

During T1 water line treatment, water lines were drained and filled with a commercially-available solution of which the main disinfecting component contained 4.0 ppm active chlorine dioxide (ClO_2_) solution (Calgonit CD-K1/K2, Calvatis GmbH, Ladenburg, Germany). The commercial solution was retained in the water lines for 24 h. Measurements of free ClO_2_ inside the waterlines were not obtained. Subsequently, the water lines were washed with the supply water by continuous flushing for 10 min. Under normal operating conditions. The T2 water line was performed by continuous pumping of acidic cleaner containing 3.0% peroxyacetic acid (PAA) and hydrogen peroxide (Calgonit DS 625, Calvatis GmbH, Ladenburg, Germany) continuously for 30 min using highpressure air pump. The water lines were then washed with the supply water and purged using a highpressure air pump until no inorganic and organic debris were visible in the water. Subsequently, the water line disinfection was performed using a commercial disinfection solution containing 4.0 ppm active ClO_2_ solution (Calgonit CD-K1/K2) which was retained in the water lines for 24 h. Subsequently, the water lines were washed with supply water by flushing for 10 min. Under normal operating conditions.

Water samples were collected by employees from the private laboratory, samples were taken from the end nipple of the drinking water line inside the vacant poultry house (HYGIENICUM GmbH, Graz, Austria). One water line was sampled at four and five poultry farms, while two water lines (line 1 and 2) were sampled at ten and nine poultry farms during T1 and T2 water line treatments (Figure 1). Two sampling timepoints were chosen, namely before treatment (BT) at the end of fattening period of 5–6 weeks, and after the water line treatment (AT) before restocking of the subsequent production cycle. As shown in Figure 1 and Supplementary Table S1, in six poultry farms during the T1 and T2 water line treatment, water samples were collected at two different sampling intervals, while other poultry farms were sampled only once. Additionally, at some poultry farms from some water lines the duplicate samples were collected, while from other poultry farms only a single sample was collected. Therefore, in total 36 (T1) and 33 (T2) BT and corresponding AT samples were collected for the microbial analysis in the present study. The water samples were collected in sterile 500 mL bottles by the private laboratory and immediately transported to the laboratory at 4°C for microbial analysis.

### 2.2 Sample processing and microbial analysis

Prior to analysis, 500 mL of water samples were centrifuged at 8000 rpm for 30 min at 4°C (Thermo Scientific, Sorvall Lynx 4000 centrifuge). All but 10 mL of the supernatant was discarded, the remainder was then resuspended using a serological 10 mL pipette (Greiner Bio One, Frickenhausen, Germany) and vortexed for 30 s.

*Campylobacter* selective enrichment and isolation were performed according to the ISO 10272-1:2006 standard for the detection of *Campylobacter* spp. in foodstuff (27). Five milliliters of the supernatant were transferred to 45 mL of Bolton broth (Thermo Fisher Scientific Ltd., Hampshire, United Kingdom) supplemented with 5% hemolyzed horse blood (Oxoid Ltd., Hampshire, United Kingdom). The Bolton broth enrichment was incubated for up to 48 h at 42°C under microaerobic conditions (10% CO_2_, 3% O_2_, 87% N_2_). After incubation modified charcoal cefoperazone deoxycholate agar (mCCDA) (Oxoid Ltd) was inoculated by fractionated loop inoculation (10 μL) and incubated at 42°C for 48 h under microaerobic conditions. Quantification of aerobic mesophilic count (AMC), *Enterobacteriaceae* (EB), and *Pseudomonadaceae* (PS) counts was carried out according to ISO reference methods (28, 29). For enumeration of AMC, EB, and PS, 5 mL of the re-suspended supernatant was transferred to 45 mL buffered peptone water (BPW) (Biokar Solabia diagnostics, Pantin Cedex, France). Subsequently, serial ten-fold dilutions were prepared up to dilution 10^−5^ in BPW (Biokar Solabia diagnostics, Pantin Cedex, France). The AMC were enumerated on trypto-caseine soy agar with 0.6% yeast extract (TSAYE) (Biokar Solabia diagnostics), while EB and PS were enumerated on red bile glucose agar (VRBG) (Merck KGaA, Darmstadt, Germany). Each dilution step (100 μL) was plated on selective agar media for the enumeration of AMC, EB, and PS counts. For dilution 10^−1^ the volume of 1 mL was divided (333 μL) on three agar plates per selective medium. Agar plates were incubated at 30°C (AMC) and 37°C (EB, PS) aerobically for up to 48 h. The EB and PS counts on VRGB agar were differentiated by their ability to ferment glucose, leading to pink colonies with or without precipitation and pale colonies for PS. Presumptive EB and PS isolates were confirmed using oxidase reaction (BioMerieux, Marcy I’Etoile, France). The minimum and maximum limits for the determination of the AMC, EB, and PS in the samples ranged between 10 and 300 CFU.

Microbial quality of water samples before (BT) and after (AT) sanitation were categorized according to AMC, EB, and PS load in two contamination levels, <4.0 log_10_ CFU/ml and ≥4.0 log_10_ CFU/ml based on existing studies (4, 7, 12).

### 2.3 Isolation and identification of bacterial and *Campylobacter* spp. isolates

The predominant bacterial colony morphologies were collected from each water sample for further confirmation. Specifically, 1–5 colonies were selected from TSAYE (*n* = 224), VRBG (*n* = 206) and mCCDA agar (*n* = 41) and then subcultured on the respective medium. The isolate list is provided in the Supplementary Table S1. The purified colonies, comprising isolates from T1 BT samples (*n* = 123), T1 AT samples (*n* = 113), T2 BT samples (*n* = 139), T2 AT samples (*n* = 96) were stored at – 80°C in brain heart infusion broth (Biokar Solabia diagnostics) supplemented with 25% (v/v) glycerol (Merck KgaA).

For DNA extraction of *Campylobacter* spp. isolates 10 μL loop of bacterial material was resuspended in 100 μL of 0.1 M Tris–HCl buffer pH 7 (Sigma Aldrich, St. Louis, MO, United States) and mixed with 400 μL Chelex® 100-Resin (BioRad, Hercules, CA, United States) (30). The bacterial Chelex® 100Resin suspension was heated at 100°C for 10 min on a block heater (Thermo Fischer Scientific Inc.), followed by short centrifugation step at 15,000 ×*g* (Eppendorf Centrifuge 5,425) for 5 s. The supernatant (100 μL) was transferred to a maximum recovery tube (Corning Incorporated Life Sciences, Reynosa, Mexico) and stored at −20°C until analysis. *Campylobacter* spp. were identified using multiplex PCR targeting genes including the conserved genus-specific 23S rRNA gene, the *Campylobacter jejuni* hippuricase gene (*hipO*) and the *Campylobacter coli* serine hydroxymethyltransferase (*glyA*) gene, as previously described (31). Briefly, a single reaction mixture (20 μL) contained diethylpyrocarbonate (DEPC) treated water (Sigma Aldrich), 1× PCR buffer, 2 mM MgCl_2_, 500 nm *hipO* forward and reverse primer, 1,000 *glyA* forward and reverse primer, 200 nm 23S forward and reverse primer, 200 μM dNTP mix, 1.5 U of Platinum Taq DNA polymerase (Platinum™ Taq DNA Polymerase, DNAfree, Thermo Fisher Scientific Inc., Waltham, MA, United States), and 5 μL template genomic DNA. The amplification was performed in T100™ Thermal Cycler (Bio-Rad, Hercules, CA, United States). The PCR cycling conditions included initial denaturation at 94°C for 2 min, 30 cycles of denaturation (94°C for 30 s), primer annealing (59°C for 30 s), elongation (72°C for 30 s) and final elongation (72°C for 7 min). The gel electrophoresis of PCRamplicons was performed in a 1.5% agarose gel containing 0.5× TrisBorateEDTA (TBE) buffer (Sigma Aldrich, St. Louis, MO, United States) and 3.5 μL peqGREEN DNA gel stain (VWR International, Radnor, United States), at 120 V for 30 min. The DNA standard Thermo Scientific™ GeneRuler™ 100 bp (Thermo Fisher Scientific Inc., Waltham, United States) was applied for fragment length comparison. We utilized the following control isolates for the DNA extraction and multiplex PCR: *C. jejuni* strain DSM 4688 and *C. coli* strain DSM 4689, obtained from Deutsche Sammlung von Mikroorganismen und Zellkulturen (DSMZ), Braunschweig, Germany.

For DNA extraction of isolates from TSAYE and VRBG, bacterial cells were lysed by boiling the suspension. A 10 μL loop of bacterial material was re-suspended in 100 μL 0.1 M Tris–HCl pH 7 buffer (Sigma Aldrich, St. Louis, MO, United States), briefly vortexed and heated at 100°C for 15 min (Thermo Scientific™ block heater, Thermo Fischer Scientific Inc.). The suspension was then centrifuged for 5 s at 15,000 ×*g* (Eppendorf Centrifuge 5,425, Hamburg, Germany) and the supernatant (70 μL) was transferred into maximum recovery tubes (Corning Incorporated Life Sciences, Reynosa, Mexico) and stored at −20°C until analysis. For identification of bacteria isolates (*n* = 471) the partial amplification of 16S rRNA gene was performed following the methods of (32, 33), using universal primer pairs 616F (5’AGAGTTTGATYMTGGCTC3′) and 1492R (5’GGYTACCT TGTTACGACTT3′) (both Microsynth AG, Blagach, Switzerland). A single PCR reaction (45 μL) contained 1× PCR buffer, 2 mM MgCl_2_, 200 nM forward and reverse primer, 250 μM dNTP mix, 2 U of Platinum Taq DNA polymerase (Platinum™ Taq DNA Polymerase, DNAfree, Thermo Fisher Scientific Inc.) and 5 μL template genomic DNA. The DNA amplification was performed in T100™ Thermal Cycler (BioRad, Hercules, CA, United States). The PCR cycling conditions included initial denaturation at 95°C for 5 min, 35 cycles of denaturation (94°C for 30 s), primer annealing (52°C for 30 s), elongation (72°C for 60 s) and final elongation (72°C for 7 min). Subsequently, the PCR amplicons were sent for purification and sanger sequencing to LGC Genomics (LGC Genomics GmbH, Berlin, Germany). The gel electrophoresis of PCR-amplicons was performed in a 1.5% agarose gel containing 0.5× TrisBorateEDTA (TBE) buffer (Sigma Aldrich, St. Louis, MO, United States) and 3.5 μL peqGREEN DNA gel stain (VWR International, Radnor, United States), at 120 V for 30 min. The DNA standard Thermo Scientific™ GeneRuler™ 100 bp (Thermo Fisher Scientific Inc., Waltham, United States) was applied for fragment length comparison. The PCR amplicons were sequenced using a 1492R (5’GGYTACCTTGTTACGACTT3′) primer. The nucleotide sequences were qualityevaluated by using Finch TV 1.4.0 (34) and MEGA X (35). The bacterial nucleotide Basic Local Alignment Search Tool (BLAST) algorithm from the National Centre for Biotechnology Information (NCBI)^1^ was used for taxonomy assignment. Sequences were assigned to genus or species level according to best matches and highest similarities (1,040 to 1,120 bp fragment length, similarity cutoff ≥97.0%). The partial rRNA gene sequence data from the isolates were deposited in the GenBank database under accession numbers MZ642358 to MZ643011.^2^ Subsequent identification of opportunistic pathogens among identified isolates was performed using the bacterial metadata base BacDive (36) and List of Prokaryotic names with Standing in Nomenclature (LPSN) (37).

### 2.4 Antimicrobial susceptibility testing

Opportunistic pathogens with clinical relevance isolated from water samples during T1 and T2 water line treatment were subjected to antimicrobial susceptibility testing (AST). The set of isolates included most frequently isolated OP, such as *Pseudomonas* spp. (*n* = 17), *Ochrobactrum* spp. (*n* = 4), *Stenotrophomonas* spp. (*n* = 3), and human relevant opportunistic pathogens including *Citrobacter* spp. (*n* = 2), *Enterobacter* spp. (*n* = 2), *Klebsiella* spp. (*n* = 1), and *Aeromonas* spp. (*n* = 1).

AST was performed for a total of 30 bacterial isolates using Sensititre™ Avian AVIAN1F Vet AST Plate (ThermoFischer Scientific Inc., Waltham, MA, United States), according to the manufacturer’s instructions. Briefly, single colonies were picked from fresh cultures grown on TSAYE for 24 h at 30°C, suspended in in sterile water to an optical density of a 0.5 McFarland standard (~ 10^8^ CFU/mL). 50 μl volumes of the bacterial suspension were transferred to wells containing different concentrations of lyophilized antimicrobials. Plates were sealed and incubated at 30°C for 24 to 48 h, after which minimum inhibitory concentrations (MIC) were read visually and defined as the lowest concentration of a given antibiotic at which no growth of the test organism was observed. *E. coli* strain ATCC 25922 was used as the internal quality control isolate. The minimum inhibitory concentration (MIC) breakpoints and definitions for multi-drug resistance (MDR; resistance to two or more antibiotic classes) (38) were determined following the standards provided by the Clinical and Laboratory Standards Institute (CLSI) manuals (39–41).

### 2.5 Data analysis

A descriptive analysis was carried out (mean, median, and standard deviation) for AMC, EB, and PS counts. The normal distribution of each data set (T1 and T2) was investigated using the Shapiro–Wilks test. Due to nonnormal distribution of data, the median values of AMC, EB, and PS counts were calculated. The Wilcoxon–Mann–Whitney rank sum test performed as a twosided test was applied to identify whether there was a significant difference between median AMC, EB and PS counts of BT and AT samples. Median AMC, EB, and PS counts in AT samples were compared for different water supplies (public vs. private), water line treatments (T1 vs. T2), following log_10_ transformation, using Wilcoxon–Mann–Whitney rank sum test. Values of *p <* 0.05 were considered as statistically significant. Statistical analyses were carried out using the R software package for statistical computing.^3^

## 3 Results

### 3.1 Aerobic mesophilic count, *Enterobacteriaceae*, and *Pseudomonadaceae* count in poultry drinking water

Ninety-nine BT samples and their corresponding AT water samples were microbiologically assessed, with a maximum acceptable microbial limit of 4.0 log_10_ CFU/ml for AMC, EB, and PS counts (Table 1). Due to non-normal distribution of the data, we used the Wilcoxon–Mann–Whitney twosided rank sum test to assess the median values for AMC, EB, and PS counts. No significant differences (*p* ≥ 0.05) were observed between the median AMC, EB, and PS counts of the BT and AT samples after T1 water line treatment (Table 1). Furthermore, we did not observe any significant difference between median AMC, EB, and PS counts in poultry farms with private and public water supply. Among the water samples, the highest median AMC counts were observed in BT (5.9 ± 1.02 log_10_ CFU/ml, median ± MAD; MAD: median absolute deviation) and AT (6.0 ± 1.17 log_10_ CFU/ml) samples. Higher median AMC counts in BT and AT samples were observed in poultry farms with a private well than those with a public water supply (Table 1). The lowest median counts were observed for EB in both BT (3.6 ± 2.13 log_10_ CFU/ml) and AT (2.3 ± 1.52 log_10_ CFU/ml) samples. In AT samples higher median EB counts were observed in poultry farms with public water supply. The PS resulted in the second highest median counts, which remained unchanged in BT (4.7 ± 1.44 log_10_ CFU/ml) and AT (4.7 ± 2.48 log_10_ CFU/ml) samples. Higher median PS counts were detected in both BT and AT samples in poultry farms with public water supply.

**Table 1:** The median aerobic mesophilic count (AMC), *Enterobacteriaceae* (EB), and *Pseudomonadaceae* (PS) in poultry drinking water samples were determined before (BT) and after waterline treatment (AT) during T1 and T2 waterline treatment using culture-dependent methods.

Treatment (T)	Water supply	Median AMC		Median AMC log_10_ ratio	Median EB		Median EB log_10_ ratio	Median PS		Median PS log_10_ ratio	*Campylobacter* spp.	
		log_10_ CFU/ml			log_10_ CFU/ml			log_10_ CFU/ml				
		BT	AT		BT	AT		BT	AT		BT	AT
1	Private	5.8 ± 1.30	5.4 ± 1.99	−0.5 ± 2.37	3.6 ± 2.13	1.6 ± 0.53	−1.1 ± 1.85	4.9 ± 1.30	3.7 ± 2.01	−0.7 ± 2.71	0/15	0/15
1	Public	5.9 ± 0.81	6.4 ± 0.88	−0.2 ± 1.68	3.5 ± 2.13	3.2 ± 2.77	−0.6 ± 1.01	4.6 ± 1.63	5.3 ± 1.95	0.3 ± 2.14	1/21	0/21
2	Private	5.0 ± 1.47	4.1 ± 0.82	−1.1 ± 1.68	1.3 ± 0.00	1.3 ± 0.00	0.0 ± 0.00	3.1 ± 2.11	2.5 ± 1.84	−1.8 ± 2.00	0/9	0/24
2	Public	4.5 ± 1.54	4.8 ± 2.15	−1.2 ± 2.88	2.6 ± 1.36	1.9 ± 0.83	−0.3 ± 3.74	3.7 ± 1.47	3.4 ± 1.54	0.8 ± 3.83	0/9	0/24
Total after T1		5.9 ± 1.02	6.0 ± 1.17	−0.2 ± 2.13	3.6 ± 2.13	2.3 ± 1.52	−0.6 ± 1.79	4.7 ± 1.44	4.7 ± 1.44	0.0 ± 2.26	1/36	0/36
Total after T2		4.6 ± 1.55	4.7 ± 1.85	−1.1 ± 2.13	2.4 ± 1.63	1.6 ± 0.42	0.0 ± 2.94	3.5 ± 1.62	3.1 ± 2.05	0.0 ± 3.12	0/33	0/33

The AMC, EB, and PS values are provided as median values (log_10_ CFU/g) and standard deviations. The presence (+) or absence (−) of *Campylobacter* spp. in water samples identified by the multiplex PCR assay. MAD, median absolute deviation.

After T1 water line treatment, high (>4.0 log_10_ CFU/ml) AMC, EB, and PS counts from BT samples decreased below the maximum acceptable microbial limit in 8/36, 7/36, and 9/36 AT samples, respectively (Supplementary Table S2). The AMC, EB, and PS below the microbial limit were observed in 1/36, 18/36, and 7/36 BT and AT samples, respectively. The AMC, EB, and PS counts above the maximum acceptable microbial limit were observed in 27/36, 11/36, and 20/36 AT samples, respectively, after T1 treatment.

During T2 water line sampling, no significant differences (*p* ≥ 0.05) were observed in the median AMC, EB, and PS counts between the BT and AT samples (Table 1). No significant difference was observed between median AMC, EB, and PS count in poultry farms with private and public water supply. The highest median counts were for AMC counts in both BT (4.6 ± 1.55 log_10_ CFU/ml) and AT (4.7 ± 1.85 log_10_ CFU/ml) samples, followed by the PS counts in BT (3.5 ± 1.62 log_10_ CFU/ml) and AT (3.1 ± 2.05 log_10_ CFU/ml) samples The lowest counts were observed in the median EB counts of BT (2.4 ± 1.63 log_10_ CFU/ml) and AT (1.6 ± 0.42 log_10_ CFU/ml) samples. Higher median AMC, EB, and PS counts were detected in AT samples in poultry farms with public water supply.

After T2 water line treatment, high (>4.0 log_10_ CFU/ml) AMC, EB, and PS counts from BT samples decreased below the maximum acceptable microbial limit in 8/33, 5/33, and 14/33 AT samples, respectively (Supplementary Table S3). The AMC, EB, and PS counts below the microbial limit were detected in 4/33, 25/33, and 10/33 samples in both BT and AT, respectively. The AMC, EB, and PS counts remained above the maximum acceptable microbial limit in 21/33, 3/33, and 9/33 AT samples, respectively, after T2 water line treatment.

The impact of T1 and T2 water line treatment on private and public water supply was evaluated by calculating the log_10_ ratio from CFU log_10_ counts detected in BT and AT water samples (Table 1). No significant differences (*p* ≥ 0.05) in log_10_ ratios were observed for AMC, EB, and PS counts after T1 and T2 water line treatment. The log_10_ ratio was not significantly different (*p* ≥ 0.05) between private and public supplied poultry farms after T1 and T2 water line treatment. The median AMC, EB, and PS ratios after T1 waterline treatment were −0.2 ± 2.13, −0.6 ± 1.79, and 0.0 ± 2.26, respectively. The analysis of log_10_ ratios after T2 waterline treatment resulted in median values of −1.1 ± 2.13 for AMC, 0.0 ± 2.94 for EB, and 0.0 ± 3.12 for PS counts. Although log_10_ ratios between poultry farms with private and public water supplies were not significantly different, we observed higher median log_10_ reduction of AMC, EB, and PS counts at poultry farms with private water supply. During T2 water line treatment higher median log_10_ reduction was observed for AMC and EB counts at poultry farms with public water supply, while higher median log_10_ reduction for PS counts was observed in poultry farms with private water supply.

**Figure 2:**
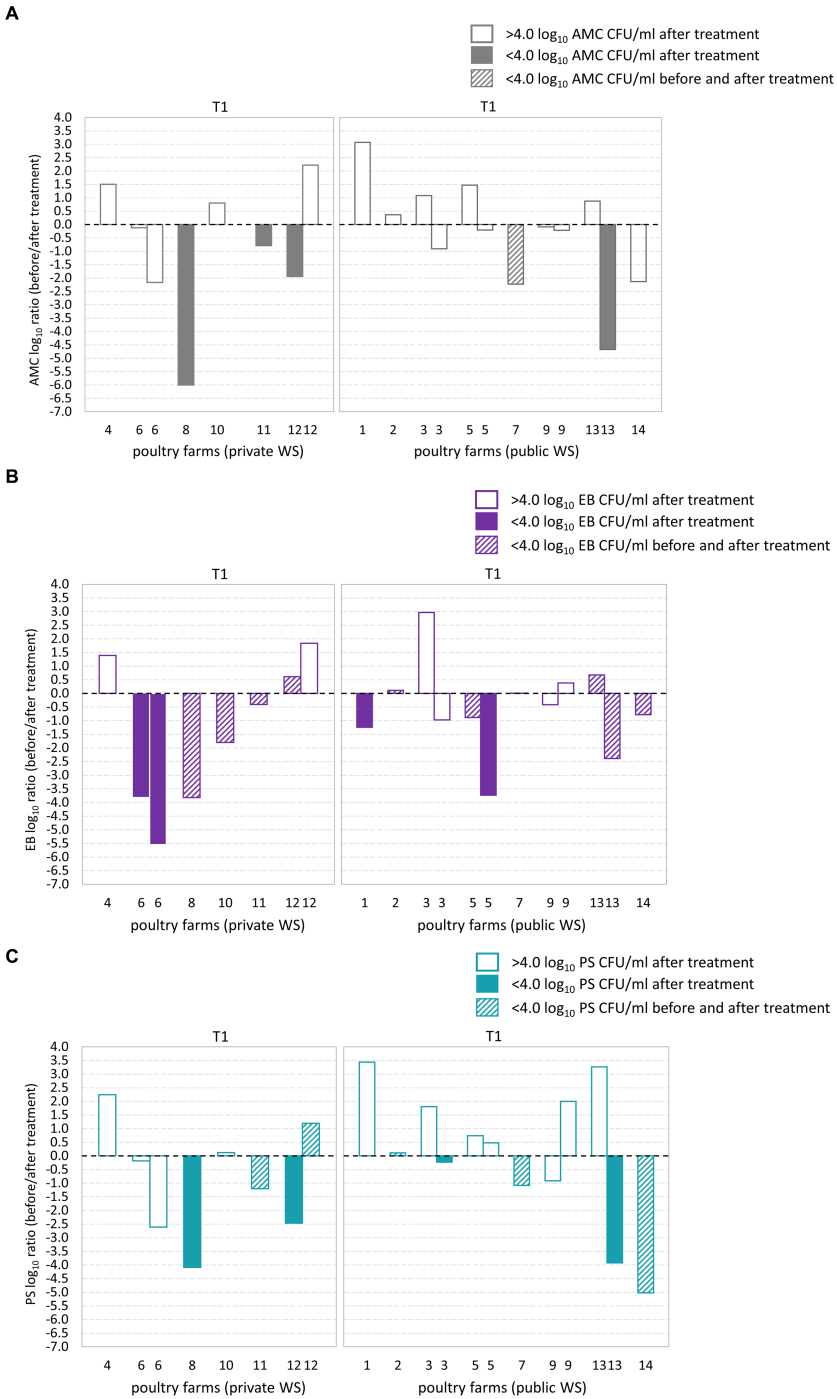
Log_10_ transformed average fold changes (before/after waterline treatment) obtained from aerobic mesophilic counts (AMC) **(A)**, *Enterobacteriaceae* (EB) **(B)**, and *Pseudomonadaceae* (PS) **(C)** in poultry drinking water. The x-axis indicates the comparison between poultry farms with private and public water supply (WS) after T1waterline treatment. The y-axis shows the log_10_ AMC, EB, and PS count ratio. The log_10_ AMC, EB, and PS ratio was not significantly different between poultry farms with private and public water supply. No significant differences were observed in the AMC, EB, and PS log_10_ ratio after T1 waterline treatment between poultry farms with private and public WS.

Out of the 14 poultry farms assessed, five farms exhibited microbial counts below the acceptable microbial limit (<4.0 log_10_ CFU/ml) subsequent to the T1 water line treatment (Figures 2A–C). Among these farms, three had a private water supply, while the remaining two had public water supplies. Notably, poultry farm 7, which had a public water supply, exhibited an AMC count below the maximum acceptable microbial limit in both BT and corresponding AT water sample. Furthermore, 11 poultry farms exhibited EB counts below the maximum acceptable microbial limit. Of these, nine poultry farms demonstrated EB counts below the microbial limit in both BT and corresponding AT samples. Additionally, among 14 poultry farms examined, a total of eight poultry farms exhibited PS counts below the microbial limit. Out of these, four poultry farms demonstrated PS counts below the microbial limit in both BT and corresponding AT samples. Among the poultry farms that underwent two samplings, poultry farms 12 and 13 exhibited AMC and PS counts exceeding the microbial limit in one of the sampling events. Furthermore, poultry farm 12 demonstrated EB counts above the microbial limit on one of two sampling occasions.

**Figure 3:**
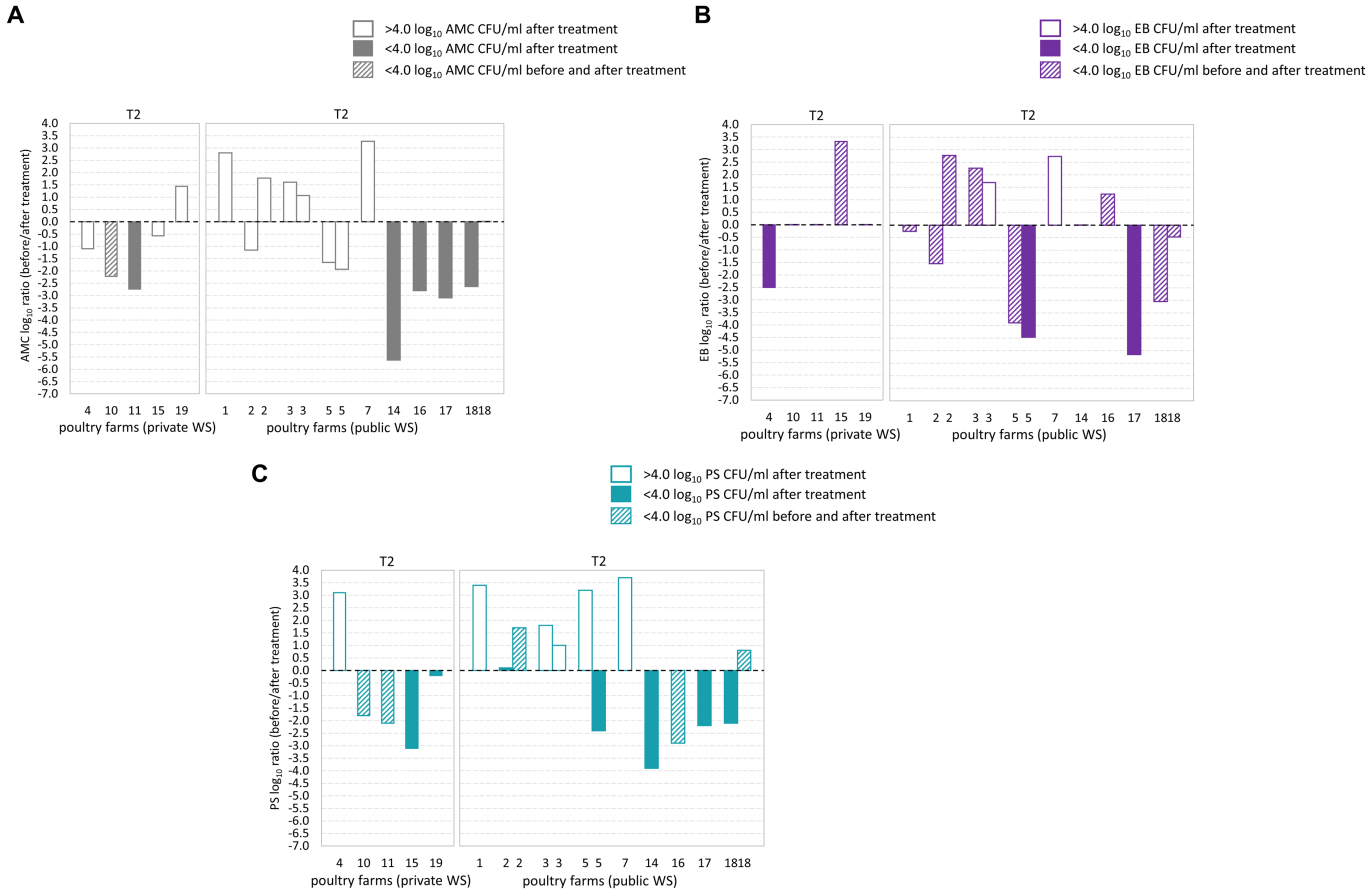
Log_10_ transformed average fold changes (before/after waterline treatment) obtained from aerobic mesophilic counts (AMC) **(A)**, *Enterobacteriaceae* (EB) **(B)**, and *Pseudomonadaceae* (PS) **(C)** in poultry drinking water. The x-axis indicates the comparison between poultry farms with private and public water supply (WS) after T2 waterline treatment. The y-axis shows the log_10_ AMC, EB, and PS count ratio. The log_10_ AMC, EB, and PS ratio was not significantly different between poultry farms with private and public water supply. No significant differences were observed in the AMC, EB, and PS log_10_ ratio after T2 waterline treatment between poultry farms with private and public WS.

During T2 waterline treatment AMC counts below the microbial limit were observed in six out of 14 poultry farms (Figures 3A–C). Of these, two poultry farms demonstrated AMC count below the microbial limit in both BT and corresponding AT samples (Figure 3B). EB counts below the microbial limit were observed in 12 out of 14 poultry farms, and among them, nine poultry farms had EB counts below the microbial limit in both the BT and corresponding AT samples. Similarly, PS counts below the microbial limit were observed in ten from 14 poultry farms, and among them, three poultry farms demonstrated PS counts below the microbial limit in BT and corresponding AT samples. Among the poultry farms subjected to two samplings, poultry farm 18 demonstrated AMC, PS and EB counts below the microbial limit during one of the sampling occasions. However, after second sampling, the AMC load in water samples exceeded the microbial limit. Notably, the PS and EB counts remained below the microbial limit during both sampling occasions.

### 3.2 Bacterial isolate identification in poultry drinking water

Isolate taxonomic assignment was performed using partial sequencing of 16S rRNA gene. In the present study, isolate sequences showed ≥97.0% similarity to the reference sequence in the NCBI database. In BT samples, 123 isolates corresponded to 24 genera and 55 species, while in AT samples, the 113 isolates corresponded to 22 genera and 40 species. Further analysis of bacterial isolates revealed that in BT and AT samples, 43.1% (*n* = 41 isolates) and 36.3% (*n* = 53 isolates) of sequenced isolates were assigned to OP, found in 29/36 BT and 17/36 AT samples (Table 2). The isolates from BT samples contained OP represented by 16 genera and 19 species, while isolates from AT samples contained OP represented by 12 genera and 12 species OP. Furthermore, *C. jejuni* was detected using multiplex PCR in a single BT water sample from a poultry farm with a public water supply.

**Table 2:** An overview of the number of isolate sequences assigned to the different phyla and genera using similarity cut-off of ≥97.0% after partial sequencing of 16S rRNA gene.

	T1		T2	
	Isolate diversity		Isolate diversity	
Sampling timepoint and isolate number	BT (*n* = 123)	AT (*n* = 113)	BT (*n* = 139)	AT (*n* = 93)
	*n*	*n*	*n*	*n*
Phylum	4	3	4	3
Genus	24	18	26	21
Opportunistic pathogens (≥97.0% sequence similarity)	*n*=53	53	43	33

The assigned bacterial isolate sequences encompass the classification of opportunistic pathogens present in water samples collected before (BT) and after (AT) the T1 and T2 water line treatment.

During the T2 water line treatment, 139 isolates in the BT corresponded to 26 genera and 46 species, whereas 96 isolates in AT samples corresponded to 21 genera and 33 species (Table 2). Among the sequenced isolates, 30.9% (*n* = 43 isolates) and 33.3% (*n* = 33 isolates) corresponded to OP, isolated from 20/33 BT and 14/33 AT samples, respectively. The OP in the BT samples comprised 10 genera, and 14 species, while the OP in the AT samples comprised 11 genera and 14 species. No *Campylobacter* spp. were detected in poultry drinking water samples during the T2 water line treatment.

**Figure 4:**
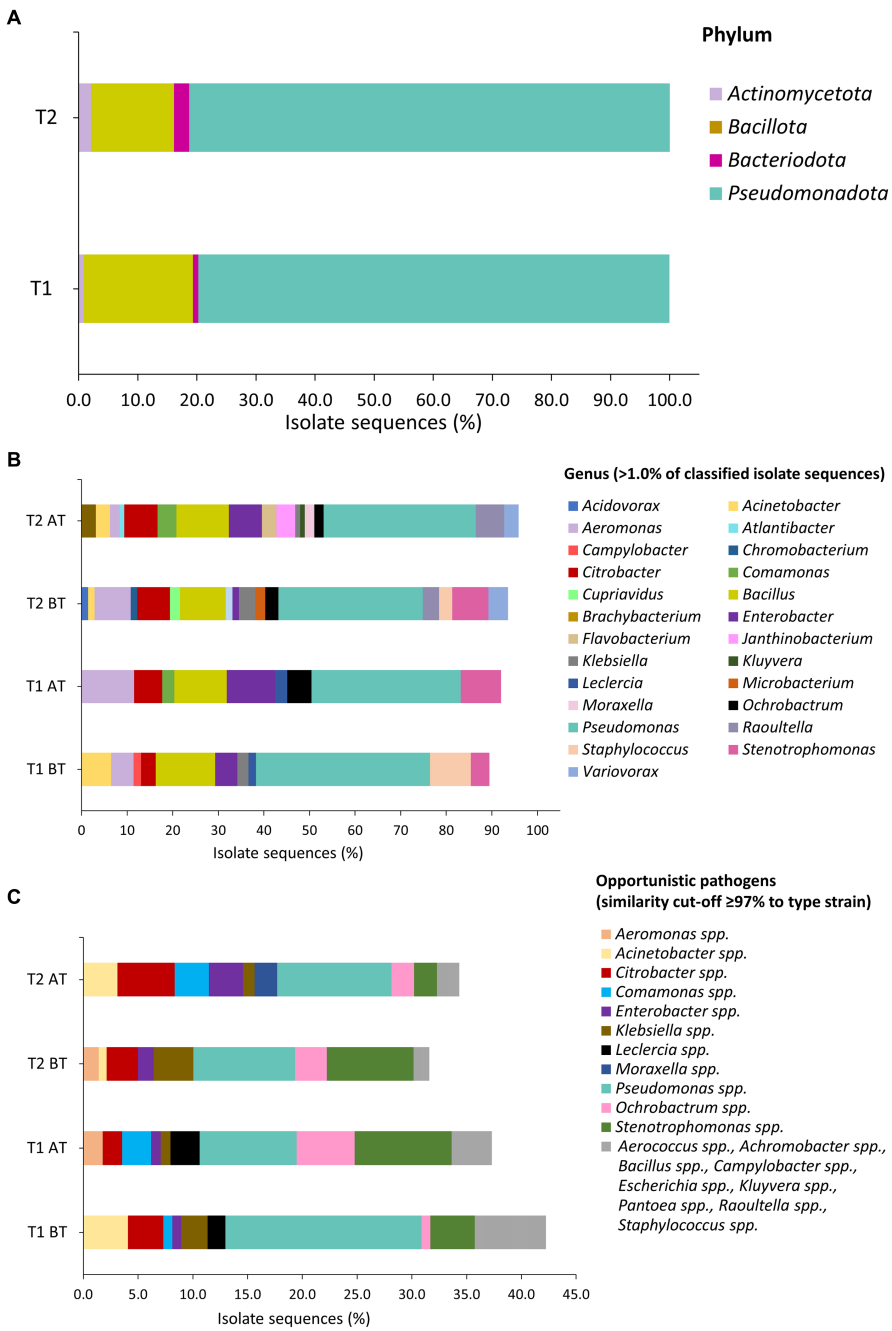
Taxonomic classification of isolates based on partial sequencing of 16S rRNA gene on phylum **(A)**, genus **(B)**, and opportunistic pathogens **(C)** level in water samples during T1 and T2 waterline treatments. Sequence similarity cut-off of ≥97.0% was applied for assignment of isolate sequences (1,040 to 1,120 bp fragment) to type strain was applied. **(C)** The bacterial sequences that were isolated from water samples one to two times are indicated by the grey color.

Figures 4A,B represents the taxonomic classification of assigned isolate sequences at phylum, and genus level. The predominant phyla in BT and AT samples were *Pseudomonadota,* followed by *Bacillota, Actinomycetota*, and *Bacteroidota* (Figure 4A). The frequently isolated genera during both T1 and T2 water line treatment in BT and AT samples were *Aeromonas, Bacillus, Citrobacter, Enterobacter, Pseudomonas* and *Stenotrophomonas* (Figure 4B). Among these, *Pseudomonas* (BT 38.2%; AT 32.7%) and *Bacillus* (BT, 13.0%, AT, 11.5%) were most commonly observed genera during T1 water line treatment. Similarly, during T2 water line treatment, *Pseudomonas* (BT, 31.7%; AT, 33.3%) and *Bacillus* (BT, 10.1%; AT, 11.5%) were predominant genera in BT and AT samples. The Figure 4B depicts the percentage identification of other observed genera during T1 and T2 water line treatments. The majority of sequenced isolates classified as OP in BT and AT samples during T1 and T2 water belonged to the *Pseudomonas* spp., followed by *Stenotrophomonas* spp., *Citrobacter* spp., *Ochrobactrum* spp., and *Acinetobacter* spp. (Figure 4C). Furthermore, isolates of *Enterobacter* spp. and *Klebsiella* spp. genera were isolated during T1 and T2 sampling. The *Pseudomonas* spp. isolates identified as OP were most frequently observed bacteria sequences during both T1 (BT, 22/123 isolates; AT, 10/113 isolates) and T2 (BT, 13/139 isolates; AT, 10/96 isolates) sampling.

Before and after the T1 water line treatment, *Pseudomonas* spp. was isolated from BT and AT samples in 12/14 and 9/14 poultry farms, respectively (Table 3). Isolate sequences of OPs were detected in BT samples of 11 out of 14 poultry farms and in AT samples of 9 out of 14 poultry farms. Among the frequently observed genera before and after T2 treatment, the genus *Pseudomonas* was isolated from the BT and AT samples in 12 out of 14 poultry farms and 9 out of 14 poultry farms, respectively (Table 4). The OP were observed in 10 out of 14 poultry farms in BT samples and in 9 out of 14 poultry farms in AT samples after T2 water line treatment.

**Table 3:** The isolate diversity in poultry drinking water samples was assessed using partial sequencing of 16S rRNA gene of cultured isolates collected during chemical waterline treatment with 4.0 ppm active ClO_2_ waterline treatment (T1) at poultry farms.

		Waterline treatment (T1)														Per sample isolation	
	Water supply	Private	Private	Private	Private	Private	Private	Public	Public	Public	Public	Public	Public	Public	Public	BT (*n* = 36)	AT (*n* = 36)
	Poultry farm	4	6	8	10	11	12	1	2	3	5	7	9	13	14		
	Water sample	BT/AT	BT/AT	BT/AT	BT/AT	BT/AT	BT/AT	BT/AT	BT/AT	BT/AT	BT/AT	BT/AT	BT/AT	BT/AT	BT/AT		
Phylum (*n* = 4)	Genus (*n* = 29)																
*Pseudomonadota* (*n* = 20)	*Achromobacter*												1/1^1^			1	1
	*Acinetobacter*	1/0	1/0	2/0					4/0							5	0
	*Aeromonas*	0/1			0/1			1/0	2/9	1/0	0/2			2/0		5	5
	*Atlantibacter*													0/1		0	1
	*Campylobacter*													2/0		1	0
	*Citrobacter*									3/5	1/0		2/0			2	3
	*Comamonas*		0/2				1/0						0/1			1	2
	*Enterobacter*		2/0				1/1			0/1			2/10		1/0	4	6
	*Escherichia*		0/1													0	1
	*Klebsiella*									2/1	1/0					2	1
	*Kluyvera*				1/0											1	0
	*Leclercia*			1/0			1/3									2	1
	*Ochrobactrum*				0/1				0/5						1/0	1	3
	*Pantonea*				0/1											0	1
	*Phytobacter*												0/1		1/0	1	1
	*Pigmentiphaga*							1/0								1	0
	*Pseudomonas*	3/3	10/7		2/2	3/0	3/7	2/2	3/0	2/6	7/6	2/0	7/1	3/3		26	18
	*Raoultella*		1/0													1	0
	*Rhizobium*													0/1		0	1
	*Stenotrophomonas*			1/0			3/1		0/1		0/5		3/2	1/1	1/0	5	10
*Actinomycetota* (*n* = 2)	*Brachybacterium*													1/0		1	0
	*Microbacterium*			1/0												1	0
*Bacillota* (*n* = 5)	*Aerococcus*	1/0														1	0
	*Bacillus*			1/0	1/0	2/2			4/6	4/1	4/3				0/1	10	7
	*Lysinibacillus*						1/0									1	0
	*Planococcus*								1/0							1	0
	*Staphylococcus*		1/0		1/1		3/0		1/0					3/0	2/0	10	1
*Bacteroidota* (*n* = 2)	*Chryseobacterium*				0/1											0	1
	*Sphingobacterium*		1/0													1	0
Bacterial diversity on poultry farm		3/2	6/3	5/0	4/6	2/1	7/4	3/1	6/4	5/5	4/4	1/0	5/6	6/4	5/1		
Identified opportunistic pathogens		1/0	4/1	0/0	2/1	0/0	4/3	1/1	2/2	3/2	4/3	0/0	4/3	1/1	0/0	29/36	17/36

The occurrence of each genus in sample collected before treatment (BT, *n* = 36) and after treatment (AT, *n* = 36) was determined. The percentage of isolate occurrence was calculated based on cultured isolates from BT (*n* = 123) and AT (*n* = 113) samples. The isolate diversity at each poultry farm was evaluated in both BT and AT samples, and the presence of opportunistic pathogens was also determined. ^1^Number of bacterial isolates isolated from BT and AT samples.

**Table 4:** The isolate diversity in poultry drinking water samples was assessed using partial sequencing of 16S rRNA gene of cultured isolates collected during combined chemical (3.0% peroxyacetic acid [PAA] and 4.0 ppm active ClO_2_) with mechanical (purging of waterlines with a high-pressure air pump) waterline treatment (T2) in poultry farms.

		Waterline treatment (T2)														Per sample isolation	
	Water supply	Private	Private	Private	Private	Private	Public	Public	Public	Public	Public	Public	Public	Public	Public	BS (*n* = 33)	AS (*n* = 33)
	Poultry farms	4	10	11	15	19	1	2	3	5	7	14	16	17	18		
	Water sample	BT/AT	BT/AT	BT/AT	BT/AT	BT/AT	BT/AT	BT/AT	BT/AT	BT/AT	BT/AT	BT/AT	BT/AT	BT/AT	BT/AT		
Pylum (*n* = 4)	Genus (*n* = 33)																
*Pseudomonadota* (*n* = 24)	*Acidovorax*					0/2^1^						2/0			0/1	5	4
	*Acinetobacter*			1/0			0/1	1/0		0/2						2	2
	*Aeromonas*	0/1						4/1				1/0			6/0	7	2
	*Atlantibacter*						0/1									0	1
	*Brevundomonas*				0/1											0	1
	*Buttiauxella*							1/0								1	0
	*Chromobacterium*												2/0			2	0
	*Citrobacter*	0/2			0/3		1/0	0/1	3/0	4/0	1/1				1/0	8	4
	*Comamonas*							0/4								0	3
	*Cupriavidus*			1/0	1/0								1/0			3	0
	*Enterobacter*					1/0			1/3						0/4	2	3
	*Janthinobacterium*							0/1	0/3							0	3
	*Klebsiella*				0/1		3/0			2/0						2	1
	*Kluyvera*						0/1									0	1
	*Moraxella*								0/2							0	1
	*Ochrobactrum*							2/2							2/0	3	1
	*Pantonea*							0/2								0	1
	*Phytobacter*						1/0									1	0
	*Pigmentiphaga*														1/0	1	0
	*Pseudaeromonas*												1/0			1	0
	*Pseudomonas*	1/1	5/0	1/0	6/0	4/1	0/2	2/1	0/8	5/6	3/3	5/0	1/0	4/3	7/7	19	16
	*Raoultella*							1/0					4/0			3	0
	*Stenotrophomonas*			2/0	1/0	2/1			6/0	0/5						7	3
	*Variovorax*	2/0		2/0	1/0	0/1									1/2	5	2
*Actinomycetota* (*n* = 2)	*Brachybacterium*													2/0		1	0
	*Microbacterium*	1/0		1/0											1/0	3	0
*Bacilliota* (*n* = 4)	*Bacillus*	1/1		3/0		1/0		6/5	2/0	1/5						10	8
	*Jeotgalicoccus*														1/1	1	1
	*Staphylococcus*							1/0						2/0	1/1	3	1
	*Trichococcus*									1/0						1	0
*Bacteroidota* (*n* = 3)	*Chryseobacterium*			1/0												1	0
	*Flavobacterium*						0/1		0/2			1/0				1	3
	*Pedobacter*									1/0						1	0
Bacterial diversity on poultry farm		4/4	1/0	9/0	4/3	4/4	3/5	8/8	4/5	6/4	2/2	4/0	5/0	3/1	9/6		
Identified opportunistic pathogens		0/1	0/0	2/0	1/1	2/0	1/1	2/2	3/2	3/2	1/1	1/0	0/0	1/1	2/3	19/33	14/33

The occurrence of each genus in sample collected before treatment (BT, *n* = 33) and after treatment (AT, *n* = 33) was determined. The percentage of isolate occurrence was calculated based on cultured isolates from BT (*n* = 139) and AT (*n* = 96) samples. The isolate diversity at each poultry farm was evaluated in both BT and AT samples, and the presence of opportunistic pathogens was also determined. ^1^Number of bacterial isolates isolated from BT and AT samples.

### 3.3 Antibiotic susceptibility patterns of bacterial isolates obtained from poultry drinking water

The susceptibility of bacterial isolates recovered from BT (*n* = 14) and AT (*n* = 16) water samples during T1 and T2 water line treatments to 18 antibiotic agents commonly used in poultry production was evaluated using Avian AVIAN1F Vet AST susceptibility plates (Table 5). The goal was to investigate AMR in the most frequently isolated OP isolates, including isolates belonging to *Pseudomonas* spp., *Stenotrophomonas* spp., *Ochrobactrum* spp., as well as AMR in specific waterborne OP important to human health, such as *Aeromonas* spp., *Citrobacter* spp., *Enterobacter* spp., and *Klebsiella* spp.

**Table 5:** Antimicrobial resistance among bacterial isolates before (BT) and after (AT) waterline treatment to a panel of veterinary antimicrobials commonly used in the poultry production.

				Antimicrobial class^1^ (in μg/ml):										
				Aminoglycosides				Fluoroquinolones	Cephalosporins	Tetracyclines	Phenicols	Sulfonamides		Diaminopyrimidine/sulfonamides
Opportunistic pathogens^2^	Treatment^3^	Time-poin^4^	Isolates (*n*)	GEN	SPE	NEO	STR	ENR	XNL	TET and OXY	FFN	SDM	STZ	SXT
				≥8	≥64	≥32	≥1,024	≥2/1	≥4	≥8	≥8	≥256		≥2/38
*Citrobacter* spp.	1	BT	1	0/1	1/1	1/1	0/1	1/1	0/1	1/1	1/1	1/1	1/1	1/1
	2	AT	1	0/1	1/1	1/1	0/1	1/1	0/1	1/1	1/1	1/1	1/1	1/1
*Enterobacter* spp.	1	AT	1	0/1	0/1	0/1	0/1	0/1	1/1	1/1	1/1	1/1	0/1	0/1
	2	AT	1	0/1	0/1	0/1	0/1	0/1	0/1	1/1	0/1	1/1	0/1	0/1
*Klebsiella* spp.	2	BT	1	1/1	1/1	1/1	0/1	0/1	0/1	0/1	0/1	1/1	1/1	0/1
*Ochrobactrum* spp.	1	AT	1	0/1	1/1	1/1	0/1	0/1	1/1	0/1	0/1	1/1	0/1	0/1
	2	BT	2	0/2	2/2	2/2	0/2	0/2	2/2	0/2	0/2	2/2	0/2	0/2
	2	AT	1	0/1	1/1	1/1	0/1	0/1	1/1	0/1	0/1	1/1	0/1	0/1
*Pseudomonas* spp.	1	BT	8	0/8	8/8	0/8	1/8	3/7	8/8	NA^5^	8/8	8/8	1/8	NA
	1	AT	6	0/6	6/6	6/6	2/6	2/6	2/6		6/6	6/6	1/6	
	2	BT	1	1/1	1/1	0/1	0/1	0/1	1/1		1/1	1/1	0/1	
	2	AT	2	0/2	2/2	0/2	1/2	0/2	2/2		2/2	2/2	0/2	
*Aeromonas* spp.	2	AT	1	0/1	0/1	0/1	0/1	0/1	0/1	0/1	0/1	0/1	0/1	0/1
*Stenotrophomonas* spp.	1	AT	1	1/1	1/1	1/1	1/1	0/1	1/1	NA	0/1	0/1	0/1	0/1
	2	BT	1	0/1	1/1	1/1	0/1	0/1	1/1		0/1	1/1	1/1	1/1
	2	AT	1	1/1	1/1	1/1	0/1	0/1	1/1		0/1	0/1	0/1	0/1
BT (*n*/*N*)^6^			14	2/14	14/14	5/14	1/14	4/14	12/14	1/14	10/14	14/14	4/14	2/14
AT (*n*/*N*)			16	2/16	13/16	11/16	4/16	4/16	13/16	3/16	10/16	13/16	2/16	1/16
Total (*n*/*N*)			30	4/30	27/30	16/30	5/30	7/30	25/30	4/10	20/30	27/30	6/30	3/30

^1^The resistance breakpoints for selected antimicrobial classes represented by the antimicrobial agents in μg/ml for ≥8 gentamicin (GEN); ≥64 spectinomycin (SPE), ≥32 neomycin (NEO); ≥1,024 streptomycin (STR); ≥2/1 enrofloxacin (ENR); ≥4 ceftiofur (XNL); ≥8 tetracycline (TET) and oxytetracycline (OXY); ≥8 florfenicol (FFN); ≥256 sulfadimethoxine (SDM) and sulfathiazole (STZ); ≥2/38 trimethoprim-sulfamethoxazole (STX). ^2^Bacteria species identified by partial sequencing of 16S rRNA gene. ^3^Waterline treatment type. ^4^Isolate identification in water sample before treatment (BT) and after treatment. ^5^NA: not applicable, bacteria have intrinsic resistance against the antimicrobial agent. ^6^*n*/*N*: number of isolates resistant to particular antimicrobial agent/total isolates tested.

The highest level of AMR was observed against spectinomycin and sulfadimethoxin (90.0%; 27/30 isolates each), followed by ceftiofur (83.3%; 25/30 isolates), florfenicol (66.6%; 20/30 isolates), and neomycin (53.5%, 16/30 isolates). Further, some isolates were resistant to enrofloxacin (23.3%; 13/30 isolates), trimethoprim-sulfamethoxazole (23.1%; 3/13 isolates), sulfathiazole (20.0%; 6/30 isolates), streptomycin (16.7%, 5/30 isolates), gentamicin (13.3%; 4/30 isolates), and trimethoprim-sulfamethoxazole (10.0%, 3/30 isolates).

The MDR was exhibited among the isolates of *Pseudomonas* spp., (17/17 isolates), and *Stenotrophomonas* spp. (1/3 isolates), *Ochrobactrum* spp. (4/4 isolates), *Citrobacter* spp. (2/2 isolates), and *Enterobacter* spp. (1/2 isolates). All *Pseudomonas* spp. isolates showed resistance patterns exhibiting resistance to a minimum of four and a maximum of eight antibiotics. Tested *Stenotrophomonas* spp. isolates also demonstrated resistance patterns to a minimum of four and a maximum of six antibiotics. All tested *Ochrobactrum* spp. were resistant to four antibiotics. The isolates of *Citrobacter* spp. were resistant to six antimicrobial classes and nine different antibiotics. The isolates of *Enterobacter* spp. showed resistance patterns to a minimum of two and a maximum of four antibiotics. The isolates of *Klebsiella* spp. were resistant to five antibiotics, while *Aeromonas* spp. isolate was susceptible to all tested antibiotic agents.

## 4 Discussion

Providing poultry with water that meets the highest quality standards is essential to ensure the safety and quality of the products derived from these animals. The presence of high microbial loads and biofilms in the drinking water lines can have a negative effect on poultry health and performance (14). Moreover, when health issues arise within a poultry flock, antibiotics are often administered through drinking water. This practice increases the risk of antibiotic resistance within poultry farms, presenting a potential threat to both animal and human health (12).

We assessed microbial quality of poultry drinking water at the end of the drinking line based on established limits from previous studies, where AMC, EB, and PS counts below 4.0 log_10_ CFU/ml were deemed acceptable (4, 7, 12). At the end of the fattening period, AMC exceeded acceptable limits in most poultry farms tested, with similar trends observed for PS counts. However, EB remained within acceptable levels in the majority of farms. Environmental factors, such as ambient temperatures (±25°C), low water flow rates, pipeline installation type, and feed additives (often mixed with glucose) provided ample nutrients for bacteria, contributing to a high microbial load at the end of the fattening period (42). Poultry farms opt to chlorinate and/or acidify their drinking water systems due to the easy application, cost-effectiveness, and broad antimicrobial properties of these treatment systems (12). Additionally, mechanical cleaning helps remove biofilm from surfaces inside the drinking water system. Surprisingly, plate count analysis did not show a significant reduction of microbial load (AMC, EB, and PS counts) in AT samples after chemical water line treatment (T1) or combined chemical with mechanical treatment (T2). Unlike previous reports associating poultry farms with a private water supply with elevated microbial loads, we did not observe significant differences in microbial load between poultry farms with private or public water supplies (43). The microbial counts observed in our study were similar to those found on surfaces inside poultry house drinking water systems, which were typically above 6.0 log_10_ CFU (12). This suggests a limited disinfection effectiveness likely due to low concentration of applied disinfectant. Despite mechanical cleaning and subsequent disinfection, high microorganism levels persisted in the water lines, indicating that the disinfectant concentration post-mechanical treatment was insufficient to eliminate the majority of microorganisms. However, our study focused solely on microbiological parameters, overlooking vital factors such as water hardness, pH, temperature, and free ClO_2_ residues within the water lines. This limited our ability to comprehensively evaluate the efficiency of the 4 ppm active ClO_2_ and 3% PAA during water line treatments. Previous studies have highlighted the limited effectiveness of water line disinfection practices using oxidizing agents such as chlorine or hydrogen peroxide (12). This limitation primarily arises from applied concentrations being lower than recommended by suppliers, which is in alignment with our observations of high microbial load in AT samples. In addition, inconsistencies were noted in AT water samples among poultry farms that were sampled twice, emphasizing the need for frequent water quality checks in a closed system. Even with the addition of typical concentrations of hydrogen peroxide (25–50 ppm) and free chlorine (2–5 ppm) to poultry drinking water during fattening, biofilm formation was observed in minimally contaminated water (7). Therefore, regular monitoring of microbial water quality, combined with consistent water line treatment during the fattening period, is a crucial aspect of robust biosecurity programs at poultry farms. Moreover, specialized contractors have been noted to achieve more effective water line treatment compared to farmers (42, 44). Finally, Zou et al. (45) demonstrated a significant reduction of *E. coli*, *Salmonella*, *Staphylococcus aureus*, and mold in poultry drinking water after treatment with sodium dichloroisocyanurate, correlating positively with poultry health.

The presence of high microbial load in water samples led to a wide taxonomic variety among isolates in both BT and AT samples, ranging between 18 and 26 genera. While definitive taxonomic conclusions require further extensive studies, the frequent presence of genera such as *Aeromonas, Bacillus, Citrobacter, Enterobacter, Pseudomonas* and *Stenotrophomonas,* commonly associated with waste and surface waters, underscores an increased risk to both poultry and human health in this study (19, 46). Identification of genera, including *Pseudomonas, Stenotrophomonas*, and *Ochrobactrum,* were in line with the isolates found on surfaces in poultry drinking water system (12). The majority of the identified bacteria found at poultry farms independent of their water supply were OP, specifically those belonging to *Pseudomonas* spp., *Stenotrophomonas* spp., and *Ochrobactrum* spp. The OP belonging to *Pseudomonas* spp. are linked to secondary infections in both poultry and humans. In poultry, these infections can manifest as septicemia, skin lesion infections, and hemorrhagic pneumonia (47). In immunocompromised humans, they can lead to septicemia, pneumonia, and urinary tract infections (48). Previous studies have also emphasized an increased mortality rate in poultry following *P. aeruginosa* OP infection (49, 50). A previous study demonstrated enhanced adhesion to abiotic surfaces, tissue invasion through cytotoxic effects, resistance to 0.2 mg/mL chlorine, and increased AMR among *P. aeruginosa* isolates from water (51). Moreover, *Stenotrophomonas maltophilia* and *Ochrobactrum intermedium* are emerging human environmental pathogens causing infections, primarily in immunocompromised patients (52). *S. matophilia* and *P. aeruginosa* are often co-isolated from the lungs of cystic fibrosis patients, and previous research findings suggest that *S. maltophilia* modulates the virulence of *P. aeruginosa* in a multispecies biofilm (53). While *S. maltophilia* and *O. intermedium* have been recognized to cause infections in immunocompromised humans, no established link between water quality and disease development in poultry production involving these bacterial species has been reported yet. Nevertheless, notable characteristics of these bacteria, such as resistance to disinfection and heat, slow growth, and biofilm formation, emphasize the potential risk of poultry and farmer infection through direct contact with drinking water, along with the risk of crosscontamination of chicken meat products during post-slaughter processing.

During T1 water line treatment, *C. jejuni* was detected in one water sample collected before water line treatment at a poultry farm with a public water supply, while other analyzed samples tested negative. The detection of *Campylobacter* spp. in water depends on factors such as sample volume, sample number, and bacterial concentration (54, 55). Furthermore, *Campylobacter* spp. can enter a viable but nonculturable state (VBNC) under environmental stress, potentially hindering growth on conventional culture media due to limited metabolic activity (56). Consequently, *Campylobacter* spp. might have been overlooked in other analyzed water samples due to limitations in the processing method. These limitations include a small sample volume, the absence of water sample filtration, and the potential presence of *Campylobacter* spp. in the VBNC state, which cannot be detected using the ISObased methods used in the current study. While this approach may have led to missing *Campylobacter* spp., our assessment of bacterial load and diversity in the water samples examined provided a comprehensive insight into both quantitative and qualitative microbial content in poultry drinking water. Notably, previous research emphasizes that a significant presence of *Pseudomonas* spp. in poultry drinking water heightens the risk of *Campylobacter* spp. infection, as *Campylobacter* sp. isolates from poultry can persist for extended periods within *P. aeruginosa* biofilms in drinking water (57–59).

Previous studies have established poultry farms as significant reservoirs of antimicrobial resistance genes, contributing to the emergence of AMR and transmission dynamics of MDR bacteria at the humananimalenvironment interface (60–62). Our findings align with these observations, revealing MDR patterns in all tested isolates of both *Pseudomonas* spp. and *Ochrobactrum* spp. isolates from BT and AT water samples. Furthermore, a single *Stenotrophomonas* spp. from BT water sample exhibited MDR pattern. The consistent AMR patterns observed in both BT and AT water samples align with our observations of ineffective water line treatment characterized by limited disinfectant concentrations that allow for the survival and persistence of AMR bacteria within the water lines. The antimicrobials permitted for poultry treatment in Austria at the time of this study include enrofloxacin, doxycycline, trimethoprimsulfamethoxazole, amoxicillinclavulanic acid, colistin sulfate, tetracycline, and gentamicin (63–67). For the isolates we utilized in the AST, information or protocols regarding the current or past treatment of poultry on these farms were not available to the authors; therefore a detailed analysis of the potential causes of AMR in these isolates was not possible. The isolates from both BT and AT water samples exhibited increased resistance patterns to spectinomycin, sulfadimethoxin, ceftiofur, florfenicol, and neomycin, likely attributed to their widespread use in poultry health management on farms. This raises concerns, as elevated streptomycin resistance in *E. coli* isolates from broilers in several countries in Europe, including Poland, Germany, Great Britain, France and Spain was previously reported (68). Additionally, resistance to streptomycin and sulfadimethoxin was previously reported in *Salmonella* spp. isolates from poultry farms in Canada and the United States (69–72). Furthermore, these isolates exhibited resistance to ceftiofur and enrofloxacin, both of which are recognized as top priority critically important antimicrobials by the World Health Organization (73). This antimicrobial resistance raises concerns, as it can be indirectly transmitted through horizontal gene transfer to *E. coli, Salmonella* spp.*, Campylobacter* spp. and other potential poultry and human pathogens. Heinemann et al. (42) reported isolation of extendedspectrum betalactamaseproducing bacteria (ESBL) such as *P. aeruginosa*, *Enterobacter* spp., *Klebsiella* spp., and *Acinetobacter baumanni* from poultry drinking water lines and sprinkler systems. ESBL bacteria can hydrolyze extendedspectrum cephalosporins, monobactams, and penicillins and thus lead to elevated morbidity and mortality, further complicating therapeutic choices, particularly among elderly and immunocompromised individuals (74–76). The observed AMR resistance patterns in poultry drinking water isolates highlight the potential for acquiring antimicrobial resistance through wateradministered medication, posing a risk and limiting treatment options in both veterinary and human medicine (1, 42, 77–79).

The study emphasizes the persistent challenge of maintaining microbial quality in poultry drinking water. The high microbial load observed is attributed to established microbiota in the water system, resistant to suboptimal disinfectant concentrations used during cleaning. Furthermore, our findings suggest that current poultry treatment and antibiotic usage may elevate the presence of AMR bacteria in drinking water due to inefficient management. Addressing this issue necessitates regular water monitoring, consistent water line treatment, and improved farmer education. Enhancing understanding of biological processes in drinking water systems and microorganism viability can lead to better guidance on herd health and farm productivity. Identifying and mitigating onfarm water quality risks, including assessing waterline technologies affecting microbiota in drinking water and water lines, is essential for controlling pathogen and antibiotic transmission in poultry production.

## 5 Conclusion

In conclusion, the majority of poultry farms in Austria exhibited high microbial loads in drinking water, largely attributed to inadequate water line management practices, including the use of suboptimal disinfectant concentrations and inconsistent treatment. Notably, there were no significant differences observed between chemical and combined chemical and mechanical water line treatments. The prevalent microbiota in poultry included *Pseudomonas* spp., *Stenotrophomonas* spp., and *Ochrobactrum* spp. Moreover, these isolates from both before and after water line treatment samples displayed increased resistance patterns to commonly used antimicrobials to treat bacterial infections in poultry. Our results underscore the need for future studies to consider appropriate water supply management on poultry farms in terms of the One Health approach, to protect public health, and to raise awareness among farmers and veterinarians.

## Data availability statement

The datasets presented in this study can be found in online repositories. The names of the repository/repositories and accession number(s) can be found in the article/Supplementary material.

## Author contributions

AM: Data curation, Writing – original draft, Investigation, Methodology, Software, Visualization. MM: Data curation, Methodology, Writing – review & editing. KW: Data curation, Methodology, Writing – review & editing. AS: Methodology, Writing – review & editing, Investigation. IK: Investigation, Methodology, Writing – review & editing. CF: Writing – review & editing. IL: Formal analysis, Methodology, Writing – review & editing. MW: Conceptualization, Writing – review & editing. BS: Conceptualization, Data curation, Funding acquisition, Project administration, Resources, Supervision, Validation, Writing – original draft, Writing – review & editing.

## Footnotes

^1^https://blast.ncbi.nlm.nih.gov/Blast.cgi

^2^https://www.ncbi.nlm.nih.gov/genbank

^3^www.r-project.org
